# Estimating the causal effect of treatment with direct-acting antivirals on kidney function among individuals with hepatitis C virus infection

**DOI:** 10.1371/journal.pone.0268478

**Published:** 2022-05-13

**Authors:** Adrienne O’Donnell, Nathan Pham, Leandra Battisti, Rachel Epstein, David Nunes, Deirdre Sawinski, Sara Lodi

**Affiliations:** 1 Department of Biostatistics, Boston University School of Public Health, Boston, Massachusetts, United States of America; 2 Department of Gastroenterology, University of Washington, Seattle, Washington, United States of America; 3 Department of Pharmacy, Boston Medical Center, Boston, Massachusetts, United States of America; 4 Department of Medicine, Section of Infectious Diseases, Boston University School of Medicine, Boston, Massachusetts, United States of America; 5 Department of Pediatrics, Section of Infectious Diseases, Boston University School of Medicine, Boston, Massachusetts, United States of America; 6 Evans Department of Medicine, Boston University School of Medicine, Boston, Massachusetts, United States of America; 7 Nephrology and Transplant Division, Weill Cornell Medical College, New York, New York, United States of America; National Taiwan University Hospital, TAIWAN

## Abstract

**Background:**

Direct-acting antivirals (DAA) are highly effective at treating Hepatitis C virus (HCV) infection, with a cure rate >95%. However, the effect of DAAs on kidney function remains debated.

**Methods:**

We analyzed electronic health record data for DAA-naive patients with chronic HCV infection engaged in HCV care at Boston Medical Center between 2014 and 2018. We compared the following hypothetical interventions using causal inference methods: 1) initiation of DAA and 2) no DAA initiation. For patients with normal kidney function at baseline (eGFR>90 ml/min/1.73m^2^), we estimated and compared the risk for reaching Stage 3 chronic kidney disease (CKD) (eGFR≤60 ml/min/1.73m^2^) under each intervention. For patients with baseline CKD Stages 2–4 (15<eGFR≤90 ml/min/1.73m^2^), we estimated and compared the mean change in eGFR at 2 years after baseline under each intervention. We used the parametric g-formula to adjust our estimates for baseline and time-varying confounders.

**Results:**

First, among 1390 patients with normal kidney function at baseline the estimated 2-year risk difference (95% CI) of reaching Stage 3 CKD for DAA initiation versus no DAA was -1% (-3, 2). Second, among 733 patients with CKD Stage 2–4 at baseline the estimated 2-year mean difference in change in eGFR for DAA initiation versus no DAA therapy was -3 ml/min/1.73m^2^ (-8, 2).

**Conclusions:**

We found no effect of DAA initiation on kidney function, independent of baseline renal status. This suggests that DAAs may not be nephrotoxic; furthermore, in the short-term, HCV clearance may not improve CKD.

## Introduction

Hepatitis C virus (HCV) infection is a major cause of morbidity and mortality throughout the world and has important extrahepatic sequelae [1–3]. In addition to diabetes and cardiovascular disease [4], patients with HCV infection are at increased risk for chronic kidney disease (CKD) and end stage renal disease (ESRD) compared with the general population, possibly due to systematic inflammation and vascular damage [5–7].

Treatment with direct-acting antivirals (DAA) is highly effective at clearing HCV: approximately 95% of patients treated with DAAs achieve a sustained virological response (SVR) equivalent to cure [8–11]. Clinical trials have shown that an SVR following treatment with DAAs is associated with liver function improvements for patients with and without CKD [12–14]. Furthermore, treatment with DAAs has been associated with a reduction in both incidence and severity of extrahepatic manifestations of HCV infection such as diabetes and cardiovascular disease [15]. However, the effect of treatment with DAAs on kidney function remains ambiguous and needs to be further investigated.

Clinical trials that led to DAA approval generally had relatively short follow-up duration and did not estimate the mid- and long-term effect of DAA treatment on kidney function. Recent observational studies using real-world data comparing changes in estimated glomerular filtration rate (eGFR) before and after DAA treatment provide inconsistent results. Some studies observed a stabilization of or an increase in eGFR, while one demonstrated a decline in eGFR, specifically among DAA-treated individuals with normal baseline kidney function [10, 16–20]. However, current HCV guidelines do not recommend routine, frequent renal monitoring after DAA treatment in individuals with normal kidney function [21]. Therefore, studies that compare the pre- and post-DAA rate of eGFR decline could be biased by the frequency of and cause for creatinine measurements. In addition, while clinical trials of DAA therapies did not indicate renal toxicity, there have been concerns about kidney adverse effects, especially in connection with sofosbuvir, which is mainly cleared renally [20, 22, 23].

Target trial emulation is a novel approach to the design of comparative effectiveness studies [24]. With this method, the study design and statistical analysis of an observational study closely emulate the protocol components of a target trial, i.e., the ideal trial a researcher would conduct to answer the research question. We have, therefore, used the target trial approach to estimate the effect of DAA treatment on kidney function by comparing two hypothetical interventions: if all patients had initiated DAA treatment versus if all patients had not initiated DAA treatment.

## Methods

### Study data

We extracted de-identified electronic health record (EHR) data from the Boston Medical Center Clinical Data Warehouse on all patients with a record of chronic HCV infection between 2014 and 2018. Boston Medical Center is the largest safety net hospital in New England; it serves as a leading primary care provider for Boston’s vulnerable populations, including minority and low-income patients; and it has a large HCV testing and screening program [25, 26]. We collected information on inpatient, outpatient, emergency room visits; laboratory measures; prescriptions; active problem lists (i.e., clinician-compiled list of a patient’s health diagnoses); procedures; insurance status; vital status; and demographic characteristics. Chronic HCV infection was defined as having an HCV-RNA result above the laboratory level of detection or a positive HCV genotype result. We used the CKD-EPI algorithm to calculate eGFR [27, 28]. The Boston University Medical Campus Institutional Review Board approved this study. The need for participant consent was waived by the committee because the data was de-identified.

### Target trial emulation

In our real-world data, individuals with CKD or impaired kidney function tended to have more frequent renal monitoring than those with normal kidney function (S1 and S2 Figs). The differential monitoring and the potential bias due to it were important considerations. Additionally, the interpretation of changes in eGFR differs for individuals with and without CKD. Therefore, we designed two separate target trials. In Trial 1 we included patients with normal baseline kidney function, defined as eGFR>90 ml/min/1.73m^2^. In Trial 2 we included patients with CKD Stage 2–4 at baseline, defined as eGFR between 15 and 90 ml/min/1.73m^2^. The target trials were identical in all components except for the baseline eGFR inclusion criteria and the outcome definition. We then designed two cohort studies to emulate the protocols of each target trial. S1 and S2 Tables detail the target trials and trial emulations.

### Eligibility criteria

**Fig 1:**
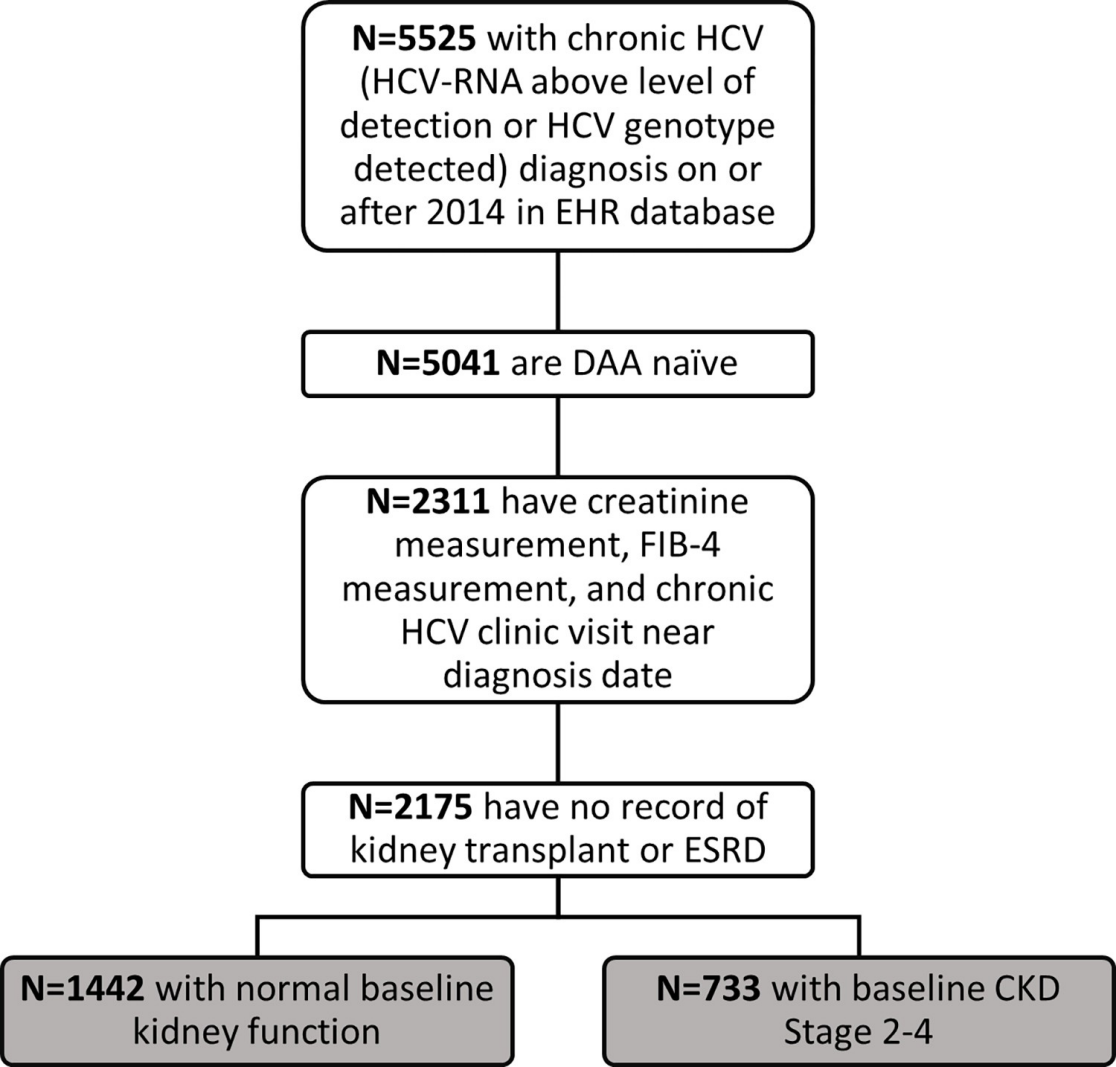
Flow chart of study participants.

Eligible individuals met the following criteria between January 2014 and December 2018: age ≥18 years, chronic HCV infection as described above, no history of kidney transplant or ESRD (defined as CKD Stage 5 or on dialysis), and the presence of a serum creatinine (to compute the baseline eGFR), transaminase, and the platelet measurements within three months of each other (to calculate a Fibrosis-4 [FIB-4] score). In addition, to capture the patients actively engaged in HCV care, we restricted eligibility to individuals with an outpatient visit for HCV infection within three months of their identified HCV infection in the EHR. Individuals who met these eligibility criteria were split into cohort 1 and cohort 2 according to their baseline eGFR and they were analyzed separately. A flow chart of these samples is presented in Fig 1.

### Interventions

For both cohorts we compared the following two DAA initiation strategies: 1) initiation of DAA within 3 months of baseline and 2) no DAA initiation. S3 Table details the DAA therapies utilized.

### Outcome

The outcome definitions differed by cohort. The outcome for cohort 1 (individuals with normal kidney function) was the development of Stage 3 CKD, defined as the first recorded eGFR≤60 ml/min/1.73m^2^.

The outcome for cohort 2 (individuals with baseline CKD Stage 2–4) was change from baseline eGFR to the last recorded eGFR between 24- and 30-months post-baseline.

### Follow-up

**Fig 2:**
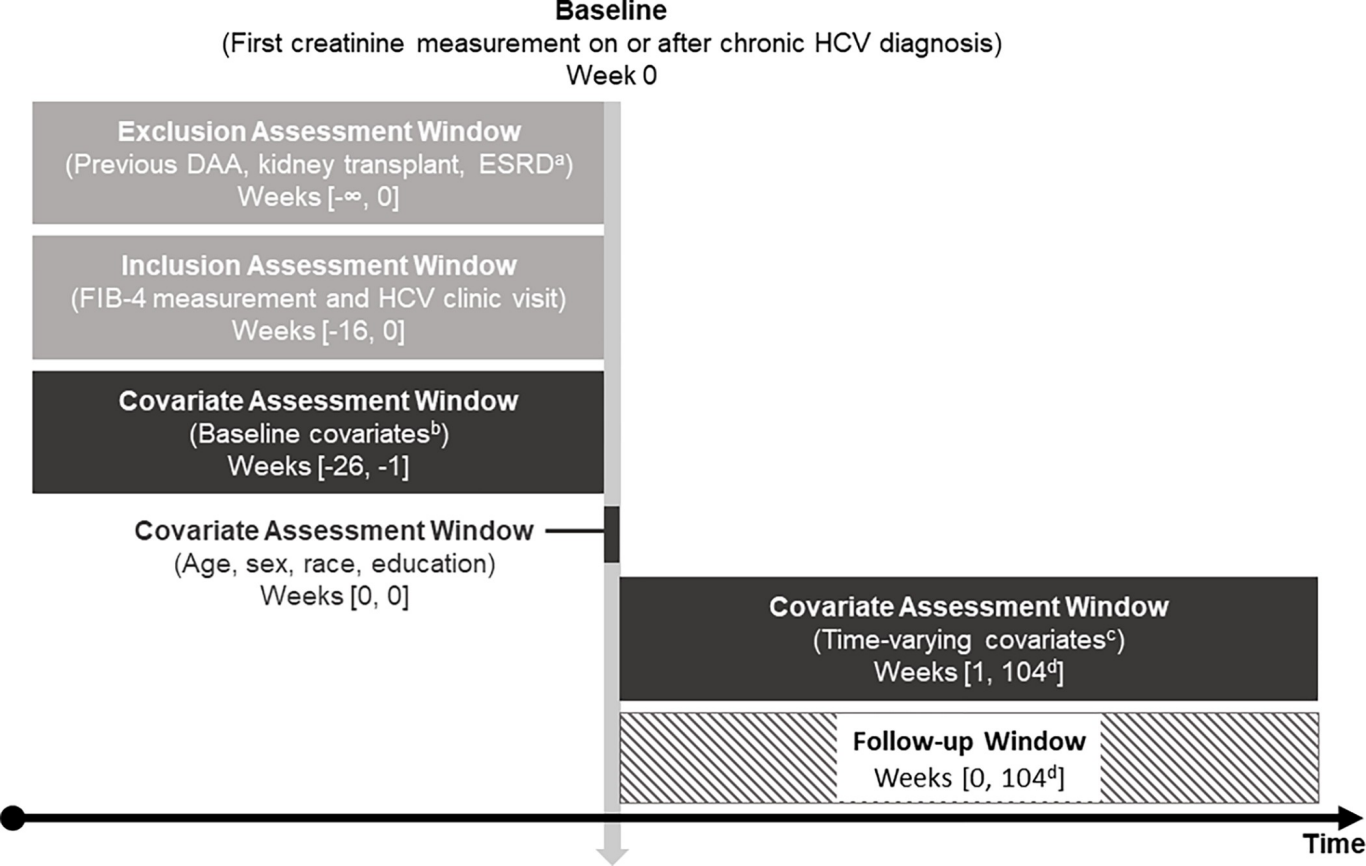
Description of cohort entry timeline. ^a^End stage renal disease (ESRD) includes dialysis and Stage 5 chronic kidney disease (CKD). ^b^Baseline covariates: Fibroscan, genotype, body mass index, insurance type, alanine transaminase, aspartate transaminase, platelets, HIV, drug or alcohol use disorder, mental illness, diabetes, hypertension. ^c^Time-varying covariates: alanine transaminase, aspartate transaminase, platelets, change in eGFR, diagnosis of drug or alcohol use disorder, mental illness, hypertension, clinic visits, treatment with DAAs. ^d^Follow-up window ends at the earliest of one of the following events: death, kidney transplant, ESRD, 104 weeks (2 years) post-baseline, administrative end of follow-up (December 2018), or, in Trial 1, date of outcome.

For both cohorts, follow-up started at baseline, defined as the earliest date all eligibility criteria were met, and ended at the earliest of one of the following events: death, kidney transplant, ESRD (defined as CKD Stage 5 or on dialysis), 2 years post-baseline, administrative end of follow-up (December 2018), or, in Trial 1, date of outcome. A summary of the study design is presented in Fig 2 [29].

### Statistical analyses

We summarized the baseline characteristics of each cohort separately using descriptive statistics.

For cohort 1, we used a time-to-event framework to estimate and compare the 2-year cumulative incidence of reaching Stage 3 CKD under the two DAA initiation strategies. For cohort 2, we estimated and compared the mean 2-year change from baseline eGFR under each intervention. Because standard statistical methods cannot appropriately adjust for time-varying confounding, we used the parametric g-formula to generate adjusted estimates [30–32]. Covariates were chosen *a priori* based on the co-authors’ clinical judgement and review of the published literature.

Like all causal inference methods, the parametric g-formula estimates the causal effect of a hypothetical intervention (here, the DAA therapy initiation) under the identifiability assumptions (positivity, exchangeability, and consistency) and assumes a correct model specification [30]. The parametric g-formula algorithm has been described in detail elsewhere [33–35]. Briefly, it works in two steps: 1) parametric regression models estimate the joint distribution of the outcome, treatment, and time-varying covariates conditional on previous treatment and covariate history and 2) the estimates in step 1 are used to simulate the distribution of the outcome under each intervention using the Monte Carlo method.

In the first step, continuous and binary time-varying covariates were modeled with separate linear and logistic regression models, respectively. All models adjusted for baseline covariates and the most recent lagged value of time-varying covariates. Baseline covariates included age, sex, race/ethnicity (Hispanic or Latino, White, Black, other), body mass index, education level, HCV genotype, insurance type, degree of liver fibrosis (by fibroscan), HIV coinfection, and diabetes. Time-varying covariates were alanine transaminase (ALT), aspartate transaminase (AST), platelet count, change from baseline eGFR, drug or alcohol use disorder [36], mental illness, outpatient visits, and hypertension. We also treated the timing of the laboratory measurements (AST, ALT, platelet count, eGFR) as potential confounders: we fit logistic regression models for these visit processes with a binary outcome of 1 if a measurement was taken at that time and 0 if not. For cohort 1, continuous eGFR was included as an additional baseline covariate, and for cohort 2 CKD stage was included as an additional baseline covariate. Skewed continuous covariates (ALT and AST) were log-transformed for normality. Details on the definition and ascertainment of covariates are presented in S4 Table.

In the second step, we used the parameter estimates from the first step to simulate the post-baseline time-varying covariates and the outcome separately under each of the two interventions. In our real-world data, eGFR is measured sporadically and long periods of time can elapse between measurements. Therefore, we imposed an eGFR measurement after 12 months in individuals without a new measurement.

We used nonparametric bootstrapping with 500 samples to obtain 95% confidence intervals (CI) around our estimates based on percentiles.

To explore the validity of our parametric assumptions, we compared the observed outcome means and time-varying covariates with those predicted by our models (S3 and S4 Figs).

All analyses were conducted in SAS version 9.4 using the publicly available GFORMULA macro [37].

### Subgroup analyses

As secondary analyses, we reran the estimation procedure in the following patient subgroups with elevated risk of CKD [38]: those with 1) type II Diabetes, 2) hypertension, and 3) age>45 years. The cut-off for age was chosen according to the bimodality of the distribution of age in the study sample (S5 Fig).

### Sensitivity analyses

We conducted several sensitivity analyses to assess the robustness of our results. First, we reran the analysis for both cohort 1 and cohort 2 excluding HCV and HIV coinfected patients [39]. Second, we reran the analysis for both cohorts restricting them to outpatient eGFR measurements only. This was done to attempt to exclude acute renal injuries. Third, we reran the analysis for cohort 1 with the follow-up extended to the database close (2018), and we reran the analysis for cohort 2 with the follow-up extended to 3 years. Fourth, we conducted the analyses with time discretized into months, instead of weeks as was done in the main analysis. Fifth, we ran a series of analyses reordering the time-varying covariates, since each parametric model conditions on past covariates. Sixth, we reran the analyses by 1) imposing an eGFR measurement after 6 months, instead of 12 months, and 2) not imposing a new eGFR measurement. In addition, for cohort 1, we ran a sensitivity analysis defining Stage 3 CKD as the first of two eGFR measurements <60 ml/min/1.73m^2^ to be consistent with the official CKD diagnosis guidelines [23]. We also reran the primary analysis additionally adjusting for concomitant use of nephrotoxic medications (nonsteroidal anti-inflammatory drugs, diuretics, and angiotensin-converting enzyme inhibitors or angiotensin receptor blockers). Finally, we reran the primary analyses to estimate the effect of treatment with Sofosbuvir-containing regimens on renal function. We did this by censoring patients treated with non-Sofosbuvir-containing regimens at their treatment start date.

## Results

Descriptive characteristics at baseline for each cohort are presented in Table 1. Descriptive characteristics stratified by treatment with DAA are presented in S5 Table. Cohort 1 included 1442 eligible patients with normal baseline kidney function. In this group, the median [IQR] age was 49 [35, 57] years, the median [IQR] eGFR was 105 ml/min/1.73m^2^ [98, 114] and 1022 (71%) were male. At baseline, 53% of patients had hypertension and 14% had diabetes. In cohort 1, 927 (64%) patients initiated DAAs during the follow-up and of these, 538 (58%) initiated within 3 months of baseline. Among the 927 patients who initiated DAAs, 822 (89%) had a post-treatment HCV-RNA measured and 792 of 822 (96%) achieved SVR defined as an undetectable HCV-RNA.

**Table 1:** Baseline characteristics stratified by cohort.

Characteristic	Cohort 1^a^ (N = 1442)	Cohort 2^b^ (N = 733)
Age, years	49 [35, 57]	59 [53, 64]
Male	1022 (71%)	435 (59%)
BMI, kg/m^2^	27 [24, 31]	28 [24, 32]
Race/ethnicity		
Black	466 (32%)	332 (45%)
Hispanic or Latino	241 (17%)	114 (16%)
White	658 (46%)	243 (33%)
Other	31 (2%)	18 (2%)
Missing	46 (3%)	26 (4%)
Health insurance		
Public	985 (68%)	486 (66%)
Private	88 (6%)	100 (14%)
Other	369 (26%)	147 (20%)
Level of education		
Less than high school	522 (36%)	325 (44%)
HS degree or more	700 (49%)	314 (43%)
Missing	220 (15%)	94 (13%)
ALT, U/L	58 [37, 108]	45 [28, 75]
AST, U/L	52 [35, 90]	45 [31, 70]
Platelet count, k/uL	209 [161, 257]	199 [157, 249]
eGFR, ml/min/1.73m^2^	105 [98, 114]	76 [64, 83]
Fibrosis Stage		
F0 –F2	431 (30%)	243 (33%)
F3 –F4	230 (16%)	163 (22%)
Missing	781 (54%)	327 (45%)
Fibrosis-4 Score Category		
<1.45 (no significant fibrosis)	694 (48%)	230 (31%)
1.45–3.25 (non-cirrhotic fibrosis)	441 (31%)	321 (44%)
>3.25 (cirrhosis)	307 (21%)	182 (25%)
Diabetes	195 (14%)	189 (26%)
Hypertension	760 (53%)	495 (68%)
Recent Diagnosis of Drug Use Disorder	628 (44%)	246 (34%)
Recent Diagnosis of Alcohol Use Disorder	158 (11%)	62 (9%)
Recent Diagnosis of Mental Illness	185 (13%)	65 (9%)
HIV co-infection	120 (8%)	88 (12%)

Median [interquartile range] or frequency (percentage) presented. BMI, body mass index. ALT, alanine transaminase. AST, aspartate transaminase.

^a^Normal kidney function defined as eGFR>90 ml/min/1.73m^2^.

^b^CKD Stage 2–4 defined as 90≥eGFR>15 ml/min/1.73m^2^.

^c^Other means such as charity or self-pay.

In cohort 1, 101 (7%) individuals developed Stage 3 CKD events and 13 died during 3,048 person-years of follow-up. As presented in Table 2, the adjusted estimated 2-year risk (95% CI) of reaching Stage 3 CKD was 5% (3%, 6%) and 5% (4%, 8%) under DAA initiation and no DAA initiation, respectively (risk difference -1% (-3%, 2%)).

**Table 2:** Estimated causal effect of DAA initiation interventions on kidney function stratified by baseline renal function.

Inclusion criteria	Intervention	Estimate^a^ (95% CI)	Estimated difference (95% CI)
Cohort 1^b^ (N = 1442)	No DAA initiation	5% (4, 8)	0 (Ref.)
	DAA initiation	5% (3, 6)	-1% (-3, 2)
Cohort 2^c^ (N = 733)	No DAA initiation	3 (-1, 8) ml/min/1.73m^2^	0 (Ref.)
	DAA initiation	0 (-3, 2) ml/min/1.73m^2^	-3 (-8, 2) ml/min/1.73m^2^

CI, confidence interval.

^a^For Cohort 1, we estimate risk of Stage 3 CKD using the parametric g-formula. For Cohort 2 we estimate mean change from baseline eGFR using the parametric g-formula.

^b^Normal kidney function defined as eGFR>90 ml/min/1.73m^2^.

^c^CKD Stage 2–4 defined as 90≥eGFR>15 ml/min/1.73m^2^.

Cohort 2 included 733 eligible patients with CKD Stage 2–4 at baseline. In this group, the median [IQR] age was 59 [53, 64] years, the median [IQR] eGFR was 76 [64, 83] ml/min/1.73m^2^ and 435 (59%) were male. At baseline, 68% had hypertension and 26% had diabetes. In cohort 2, 514 (70%) patients initiated DAA during follow-up and of these, 316 (61%) initiated DAA within 3 months of baseline. Among the 514 who initiated DAA, 494 (96%) had a post-treatment HCV-RNA measured, and 481 of 494 (97%) achieved SVR defined as an undetectable HCV-RNA.

In cohort 2, the observed mean (standard deviation, SD) change from baseline eGFR was 0 (16) ml/min/1.73m^2^. The adjusted estimated mean (95% CI) change from baseline in eGFR was 0 (-3, 2) ml/min/1.73m^2^ and 3 (-1, 8) ml/min/1.73m^2^ under DAA initiation and no DAA initiation, respectively. Compared to no DAA initiation, the mean difference was -3 (-8, 2) ml/min/1.73m^2^ under DAA initiation. There were 14 deaths during the follow-up in cohort 2.

Results of subgroup analyses are presented in S6–S8 Tables. Individuals with diabetes, with hypertension, or aged >45 years had a higher incidence of CKD Stage 3 (cohort 1) and a faster eGFR decline (cohort 2) at 2 years post-baseline compared to their counterparts without diabetes, without hypertension, or aged ≤45 years. Effect estimates were similar to those in the main analysis, although with lower precision due to smaller sample sizes. The subgroup of patients aged ≤45 years in cohort 2 had too few patients (N = 78) to allow for estimation.

Results from sensitivity analyses were consistent with main results for both cohorts (S6 and S7 Figs). The distribution of the time-varying means predicted by our models were similar to the observed means (S3 and S4 Figs).

## Discussion

We estimated the causal effect of initiating versus not initiating DAA treatment on kidney function in two cohorts of patients with HCV infection: one with normal kidney function (cohort 1) and one with baseline CKD Stage 2–4 (cohort 2). For those with normal kidney function at baseline, we observed no difference in the risk of reaching Stage 3 CKD had all patients initiated DAA therapy versus had all patients not initiated DAA therapy. Similarly, for those with CKD Stage 2–4 at baseline, we observed no difference in 2-year post-baseline change in eGFR had all versus no patients initiated DAA. These results were robust to several sensitivity analyses that varied analytic decisions and model specification. Additionally, in our secondary analysis we evaluated patient subgroups which might particularly benefit from treatment and found no effect of DAAs on renal function. Altogether these findings suggest that treatment with DAA for HCV infection neither impairs nor improves renal function over a 2-year period.

Previous observational studies estimated the association between DAA treatment and eGFR trends. Specifically, Driedger et al. reported no notable change in renal function before and after DAA initiation [17]. In another study, Medeiros et al. found no association between sofosbuvir-based DAA regimens and eGFR among a group of patients with baseline eGFR<45 ml/min/1.73m^2^, but observed improvements in renal function among those with eGFR>45 ml/min/1.73m^2^ [18]. An observational study by Sise et al. compared eGFR slope one year before and one year after DAA initiation and found no difference among those with baseline eGFR>60 ml/min/1.73m^2^ but reported improvements among those with baseline eGFR<60 ml/min/1.73m^2^ [16]. In addition, in a retrospective cohort study of patients treated with ledipasvir/sofosbuvir for HCV genotype 1b, Okubo et al. reported no changes in eGFR from pre-treatment to 12 weeks post-treatment among those with CKD Stage 3 [10]. Finally, a prospective observational study with biannual eGFR measurements found no significant difference in eGFR evolution among DAA-treated individuals who did and did not achieve SVR [19]. All these studies compared kidney function before and after DAA treatment, therefore, they were only analyzing patients who received DAA treatment. While this approach allows for control of baseline confounding factors, whereby patients act as their own controls, these estimates might be biased if patients who received DAA treatment had worse (or better) renal function than those who did not [9, 40, 41]. In contrast, we designed our study to include all patients eligible to receive DAA treatment and we adjusted for potential confounders. Despite a different study design, our results are not only consistent with the lack of a clear association between treatment with DAAs and renal function in the literature, but also are generalizable to all patients eligible to receive DAAs.

Our study has important strengths. We used longitudinal data from an urban safety net hospital with a notable HCV testing and screening program, which allowed us to include a diverse patient population. We analyzed this rich source of real-world data with causal inference methods that explicitly adjusted for time-varying confounding and selection bias. Time-varying confounding is intrinsic to this study setting: elevated liver function tests and the presence of certain covariates indicate a more pressing need for treatment, and treatment improves liver function [12, 13]. Selection bias is also present, whereby patients with CKD at baseline have more frequent post-baseline eGFR measurements than individuals with normal renal function [32]. We address both methodological issues by adjusting for variables associated with renal function, including explicit adjustment for renal monitoring frequency. By stratifying patients into cohorts according to baseline renal function, we aimed to reduce the impact of selection bias on our results. Our study explicitly estimated what would have happened had all patients initiated versus not initiated DAA, which differs from, but complements, previous studies that compared renal function before and after DAA therapy. As it would be unethical to conduct a randomized controlled trial (RCT) that assigns some patients to HCV treatment and others not, our study is as close methodologically as possible to an RCT and, notably, enables us to comment upon causation, not merely association.

Our study also has several limitations. First, our methods rely on the assumption of no residual confounding, yet we may not have captured all confounding characteristics. However, we separately analyzed two cohorts with different baseline renal function and clinical and demographic characteristics, and, thus, likely they also had different confounding structures. We found the null effect in both cohorts, therefore, it reinforced our conclusions. Second, retrospective use of EHRs is subject to error: we may have missed deaths, HCV treatments, or laboratory measurements done outside the hospital system. Third, we used prescriptions to ascertain DAA treatment initiation, but we had no record of medication adherence. Treatment completion rates for DAAs as low as 68% have been reported for HCV infected patients [11]. However, a recent study in an overlapping sub-population at Boston Medical Center found, by comparing chart-reviewed DAA adherence with EHR prescription records, that only 7% of individuals prescribed DAAs never started treatment [42]. Fourth, because of the relatively high eGFR in our sample, we used a baseline eGFR cutoff of 90 ml/min/1.73m^2^. The cutoff for clinically significant CKD is 60 ml/min/1.73m^2^, and so future studies with larger sample size and/or more renally impaired patients should replicate this research with the more stringent cutoff [21]. Finally, the majority of individuals who initiated DAA treatment received sofosbuvir-containing regimens, in line with DAA treatment in the United States during the study period. A future study with a larger database, perhaps pooling data across medical centers, may look at the effect of specific DAA combinations on renal function [20].

Despite these limitations, our results have important implications for renal monitoring among patients treated with DAA. Frequency of renal monitoring should remain the same after treatment with DAA as it was before for those with and without renal impairment. According to Kidney Disease: Improving Global Outcomes guidelines, this means at least annual eGFR monitoring for those with normal renal function and 1 to 4+ times per year for those with CKD, depending on severity [21]. Our study suggests DAAs may not be nephrotoxic and that HCV clearance may not restore renal function, but future research should continue to work to elucidate these relationships. In conclusion, we used robust methodology combined with a rich source of data and found no causal effect of DAA treatment on kidney function.

## Supporting information

S1 Fig Proportion of patients with any visit over follow-up stratified by cohort. *p<0.05, **p<0.01, ***p<0.001 for difference in proportion of patients with any visit between cohort 1 and cohort 2.
(TIF)

S2 Fig Proportion of patients with an eGFR measurement over follow-up stratified by cohort. *p<0.05, **p<0.01, ***p<0.001 for difference in proportion of patients with an eGFR measurement between cohort 1 and cohort 2.
(TIF)

S3 Fig Observed versus simulated data for cohort 1. Observed (solid line) versus simulated data (dotted line) under the natural course (i.e., treatment is not imposed). The visit process models are for the timing of the laboratory measurements.
(TIF)

S4 Fig Observed versus simulated data for cohort 2. Observed (solid line) versus simulated data (dotted line) under the natural course (i.e., treatment is not imposed). The visit process models are for the timing of the laboratory measurements.
(TIF)

S5 Fig Histogram of age distribution in study sample. (TIF)

S6 Fig Sensitivity analyses for cohort 1. Administrative end of follow-up/database close date was December 2018. Reordering covariates refers to the temporal parametric assumptions made in the model.
(TIFF)

S7 Fig Sensitivity analyses for cohort 2. Administrative end of follow-up/database close date was December 2018. Reordering covariates refers to the temporal parametric assumptions made in the model.
(TIFF)

S1 Table Cohort 1: Details of target trial and emulated trial. (DOCX)

S2 Table Cohort 2: Details of target trial and emulated trial. (DOCX)

S3 Table Direct-acting antiviral agents summary. (DOCX)

S4 Table Ascertainment of diagnoses from EHR. (DOCX)

S5 Table Descriptive characteristics stratified by observed DAA. (DOCX)

S6 Table Estimated causal effect of DAA initiation interventions on kidney function stratified by baseline renal function among those with versus without diabetes. (DOCX)

S7 Table Estimated causal effect of DAA initiation interventions on kidney function stratified by baseline renal function among those with versus without hypertension. (DOCX)

S8 Table Estimated causal effect of DAA initiation interventions on kidney function stratified by baseline renal function among those age>45 versus age≤45 years. (DOCX)
