# Supplementary figures and images for: Estimating the causal effect of treatment with direct-acting antivirals on kidney function among individuals with hepatitis C virus infection
Source: PLoS One. 2022 May 13;17(5):e0268478. doi: 10.1371/journal.pone.0268478 (PMC9106151; doi:10.1371/journal.pone.0268478)

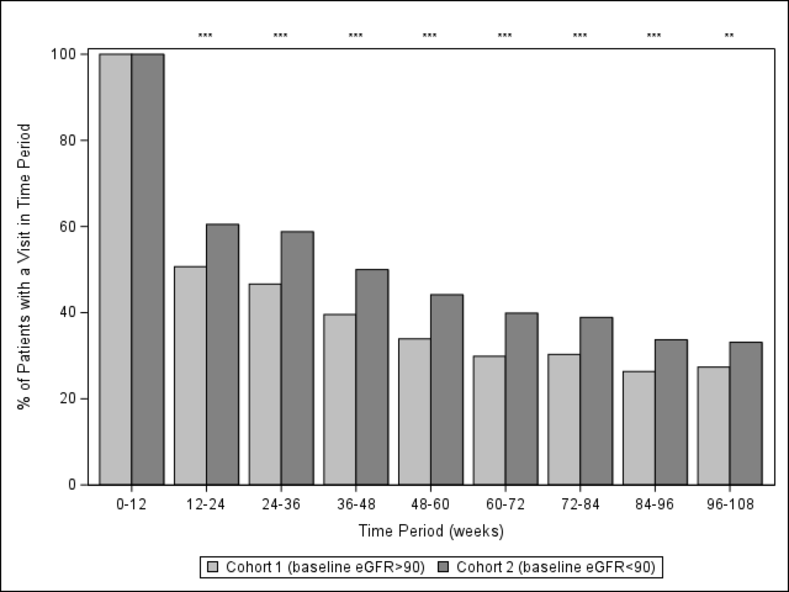

Supplement: S1 Fig — *p<0.05, **p<0.01, ***p<0.001 for difference in proportion of patients with any visit between cohort 1 and cohort 2. (TIF) [file pone.0268478.s001.tif]

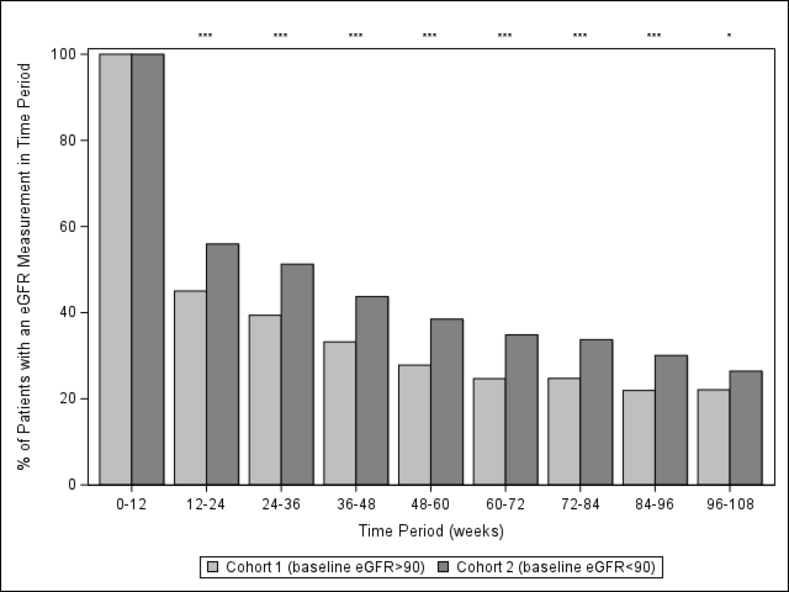

Supplement: S2 Fig — *p<0.05, **p<0.01, ***p<0.001 for difference in proportion of patients with an eGFR measurement between cohort 1 and cohort 2. (TIF) [file pone.0268478.s002.tif]

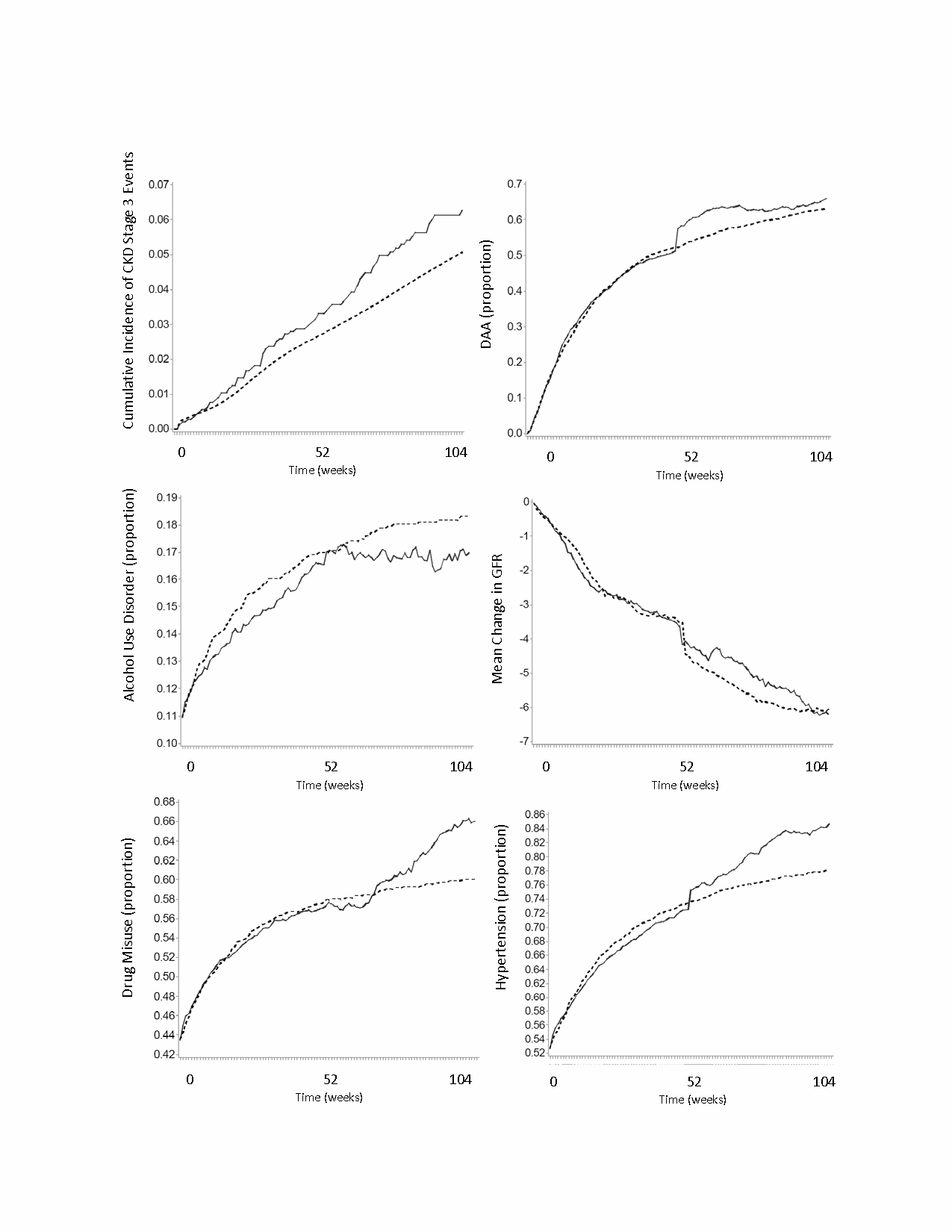

Supplement: S3 Fig — Observed (solid line) versus simulated data (dotted line) under the natural course (i.e., treatment is not imposed). The visit process models are for the timing of the laboratory measurements. (TIF) [file pone.0268478.s003.tif]

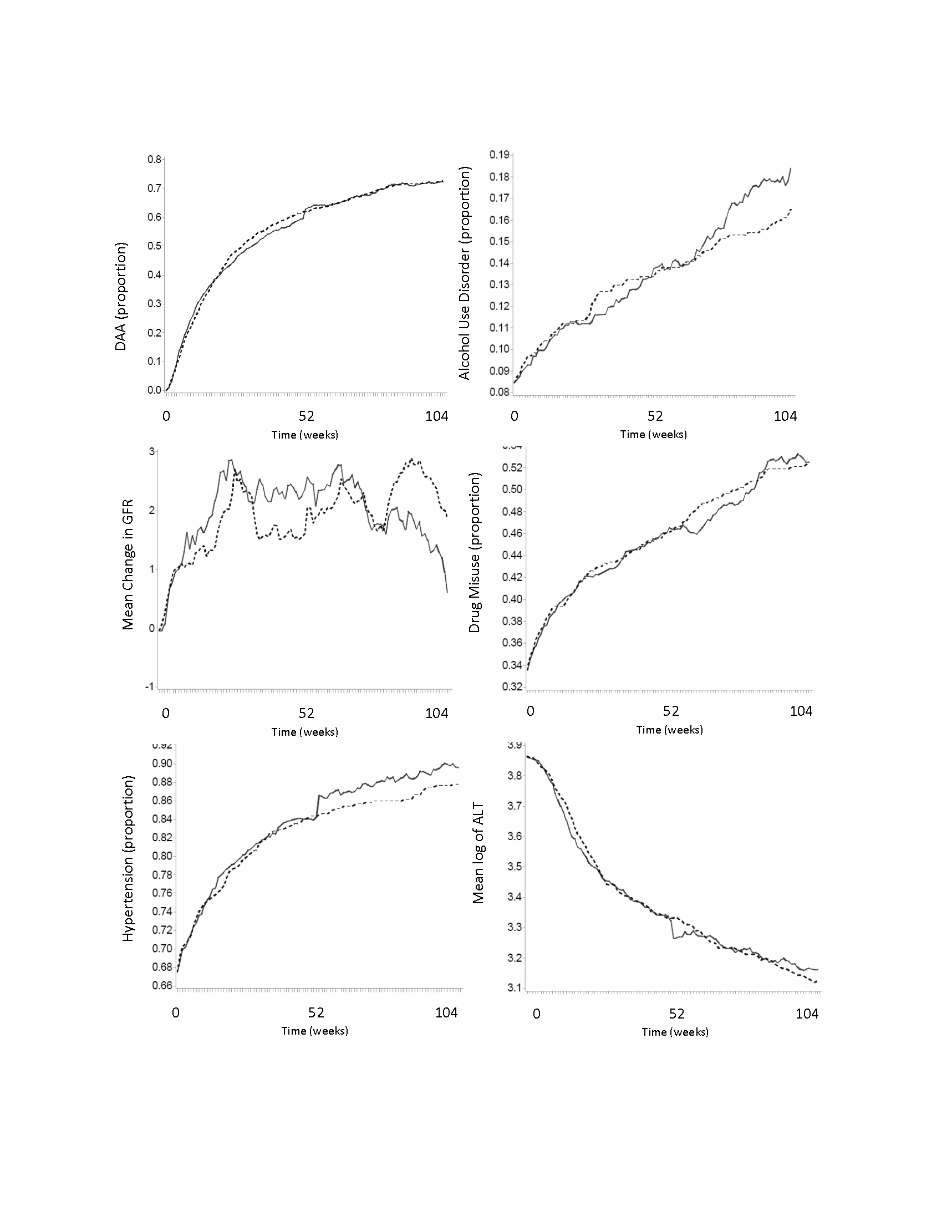

Supplement: S4 Fig — Observed (solid line) versus simulated data (dotted line) under the natural course (i.e., treatment is not imposed). The visit process models are for the timing of the laboratory measurements. (TIF) [file pone.0268478.s004.tif]

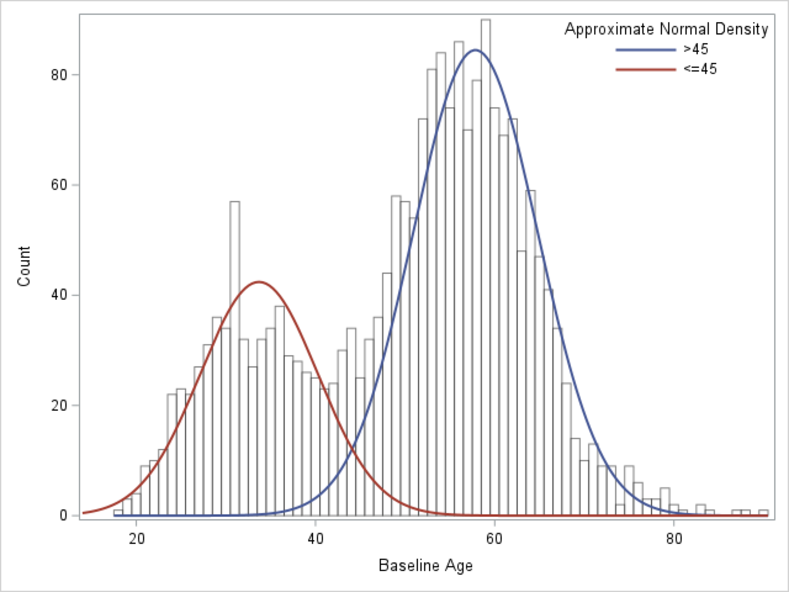

Supplement: S5 Fig — (TIF) [file pone.0268478.s005.tif]

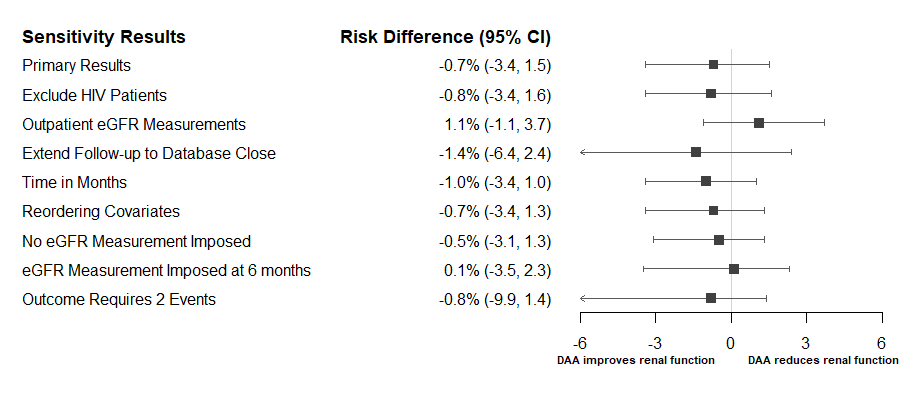

Supplement: S6 Fig — Administrative end of follow-up/database close date was December 2018. Reordering covariates refers to the temporal parametric assumptions made in the model. (TIFF) [file pone.0268478.s006.tiff]

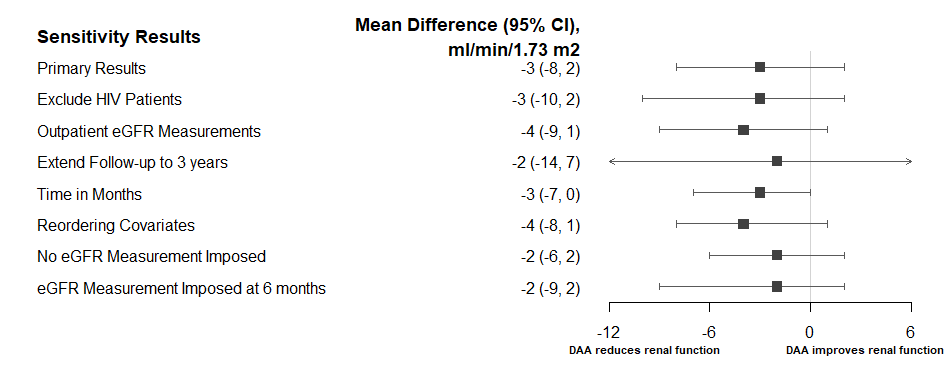

Supplement: S7 Fig — Administrative end of follow-up/database close date was December 2018. Reordering covariates refers to the temporal parametric assumptions made in the model. (TIFF) [file pone.0268478.s007.tiff]
