# Supplementary material for: Estimating the causal effect of treatment with direct-acting antivirals on kidney function among individuals with hepatitis C virus infection
Source: PLoS One. 2022 May 13;17(5):e0268478. doi: 10.1371/journal.pone.0268478 (PMC9106151; doi:10.1371/journal.pone.0268478)
Supplement: S2 Table — (DOCX) [file pone.0268478.s009.docx]

| **Component** | **Target trial** | **Emulated trial** |
| --- | --- | --- |
| Eligibility criteria | Patients must be diagnosed with chronic Hepatitis C virus after 2014, be DAA naïve, have no history of End Stage Renal Disease or kidney transplant, and have impaired renal function at baseline (defined as 90≥eGFR>15). | Same, and patients must also have creatinine and FIB-4 measurements at baseline and have accessed care at BMC for HCV. |
| Interventions | Treatment: Take DAA within 3 months of baseline.  Control: Never take DAA. | Same. |
| Treatment assignment | Patients are randomized 1-to-1 to receive treatment or control. This is an unblinded, open-label study. | Randomization is not possible, instead, identify confounders to adjust for in the analysis. |
| Outcome | Mean change in eGFR at 2 years after baseline. | Same. |
| Follow-up | Follow-up begins at randomization. Follow-up ends when a patient dies, is lost to follow-up, or at 2 years post-baseline, whichever comes first. | Follow-up begins at baseline, defined as the first date all eligibility criteria are met. Follow-up ends when a patient dies, at database close date (December 2018), or at 2 years post-baseline, whichever comes first. Follow-up may also end due to artificial censoring, whereby a patient is censored if eGFR has not been measured in 1 year. |
| Statistical analysis | The ITT effect is estimated as the mean difference in the outcome between the treatment and control groups. | The parametric g-formula is used to estimate the ITT effect as the mean difference in the outcome between the treatment and control groups, by adjusting for baseline and time-varying confounding and informative censoring. |
