# Supplementary material for: Estimating the causal effect of treatment with direct-acting antivirals on kidney function among individuals with hepatitis C virus infection
Source: PLoS One. 2022 May 13;17(5):e0268478. doi: 10.1371/journal.pone.0268478 (PMC9106151; doi:10.1371/journal.pone.0268478)
Supplement: S3 Table — (DOCX) [file pone.0268478.s010.docx]

| **Combination** | **N (%) out of 1441 DAA initiators** |
| --- | --- |
| Ledipasvir and sofosbuvir | 1007 (70%) |
| Sofosbuvir and velpatasvir | 194 (14%) |
| Sofosbuvir and ribavirin | 108 (7%) |
| Sofosbuvir and daclatasvir | 39 (3%) |
| Glecaprevir and pibrentasvir | 27 (2%) |
| Daclatasvir | <1% |
| Dasabuvir | <1% |
| Dasabuvir, ombitasvir, paritaprevir and ritonavir | <1% |
| Grazoprevir and elbasvir | <1% |
| Ledipasvir, sofosbuvir, elbasvir, and grazoprevir | <1% |
| Ledipasvir, sofosbuvir, and velpatasvir | <1% |
| Sofosbuvir and simepriver | <1% |
