# Supplementary material for: Estimating the causal effect of treatment with direct-acting antivirals on kidney function among individuals with hepatitis C virus infection
Source: PLoS One. 2022 May 13;17(5):e0268478. doi: 10.1371/journal.pone.0268478 (PMC9106151; doi:10.1371/journal.pone.0268478)
Supplement: S4 Table — (DOCX) [file pone.0268478.s011.docx]

| **Diagnosis** | **Ascertainment** |
| --- | --- |
| Chronic Hepatitis C virus infection | Detectable HCV viral load or HCV Genotype |
| Age | Years since date of birth |
| Sex | Self-report |
| BMI | Weight (in kilograms) divided by height (in meters) squared |
| Education Category | Self-report “highest grade completed” selected from: I did not attend school, 8^th^ grade or less, Some high school, Graduated high school or GED, Some College/Voc/Tech prog., Graduated college/postgrad., Other education/specify below, Declined, Unavailable  If response was “I did not attend school” or “8^th^ grade or less” or “Some high school” then Education Category was “up to high school”. If response was “Graduated high school or GED” or “Some College/Voc/Tech prog.” then Education Category was “some college”. If response was “Graduated college/postgrad.” then Education Category was “college degree or more”. |
| HCV Genotype | Genotyping was done via PCR and was categorized into 1, 2, 3, 4, or 6. For analysis, genotype was dichotomized as 1 or not 1. |
| Insurance | Most common insurance type from medical visits in the 6 months leading up to baseline. Insurance was categorized as public (e.g. government-funded such as Medicare or Medicaid), private, or other (e.g. research, charity, or self-pay). |
| Race/Ethnicity Category | Self-report race selected from: American-Indian/Native American, Asian, Black/African American, Declined/Not Available, Hispanic or Latino, Native Hawaiian/Pacific Islander, Other, Other Pacific Islander, Unknown, or White  Self-report ethnicity selected from: Hispanic or Latino, Not Hispanic or Latino  If Hispanic or Latino, then Race/Ethnicity Category is LATINX. If White, then Race/Ethnicity Category is WHITE. If Black/African American, then Race/Ethnicity Category is BLACK. Otherwise, Race/Ethnicity Category is OTHER. |
| Estimated Glomerular Filtration Rate | Serum creatinine, age, race, and gender used to estimate GFR according to the CKD-EPI equation [27]. |
| Fibroscan | METAVIR score was derived based on fibroscan stiffness. When borderline, the METAVIR score was categorized as the more severe measure (e.g. F0-F1 was grouped into the F1 category). For analysis, fibroscan was dichotomized as F0-F2 versus F3-F4. |
| Alanine transaminase | Serum ALT (U/L) |
| Aspartate transaminase | Serum AST (U/L) |
| Platelets | Platelet count (K/uL) |
| Recent Alcohol Use Disorder | First recorded relevant ICD 9/10 code: F10, Z71.4, R78.0, 790.3, T51, 305, 303, V65, 291, V69.8, 980  Follow-up starts 1 year prior to baseline. |
| Type II Diabetes | First recorded relevant ICD 9/10 code (E11, E08, E09, 249, 250 [but not 250.01 or 250.11]) or second hemoglobin A1C measurement ≥6.5 percent. |
| Recent Drug Use Disorder | First inpatient visit or second outpatient visit with relevant ICD 9/10 code: F11, F19, E850.0, 304, 305, 965.0, T40.0, T40.1, T40.2, T40.3, T40.4, T40.6, T40.7, T40.8, T40.9, F16, F12, T52.9, F18, R78.1, 970, F14, T40.5, R78.2, 969.7, F15, T43.6, 969.4, F13, T42.3, T42.4, T42.5, T42.6, T42.7, T42.8, 292, 648.3, 796.0, 962.1, 965.8, 966.3, 966.4, 967.0, 968.4, 968.5, 969.6, 969.8, 969.9, 970, V65.42, Z71.5, Z50.3, T42, T38.7, T40.8, T40.9, T41.2, T43.6, T43.7, T43.8, T43.9, T50.7, 292.^32^  Follow-up starts 1 year prior to baseline. |
| End Stage Renal Disease | Defined as CKD Stage 5 or on dialysis.  First record with relevant ICD 9/10 code (585.5, 585.6, V45.11, N18.6, 54.98, T85, Z99.2, T82, Z49, T81) or eGFR<15 or dialysis (hemodialysis and peritoneal dialysis). |
| Kidney transplant | First record with relevant ICD 9/10 code (Z94.0, 55.6, 355.6, 455.6, 755.6, Q55.6, V55.6, T86.1, Z48.22) or medication indicating kidney transplant (cyclosporine or tacrolimus) or direct record of kidney transplant |
| HIV | First record with relevant ICD 9/10 code (042, B20, Z21, V08). |
| Hypertension | First record with relevant ICD 9/10 code (401.0, 401.1, 401.9, I10) or elevated blood pressure (systolic blood pressure>140 mmHg or diastolic blood pressure >90mmHg) or antihypertensive medication (Lisinopril, Enalapril, Losartan, Valsartan, Amlodipine, Nifedipine, Hydralazine, Carvedilol, Labetalol, Atenolol, Hydrochlorothiazide, Chlorothiazide). |
| Recent Mental Illness | Depression, bipolar disorder, or schizophrenia.  First record with relevant ICD 9/10 code: F32, F31, F20, F25, F33, F96, F95, 309, 311, 648. |
| DAA | Antiviral medications for HCV categorized as direct-acting antiviral agents or interferon-based therapies. First record of DAA was used as DAA start date. |

Abbreviations: HCV=hepatitis C virus. BMI=body mass index. GED=general education development. PCR=Polymerase Chain Reaction. GFR=glomerular filtration rate. CKD-EPI=Chronic Kidney Disease: Epidemiology Collaboration. METAVIR= Meta-analysis of Histological Data in Viral Hepatitis. ALT=alanine transaminase. AST=aspartate transaminase. ICD=International Classification of Diseases. DAA=direct-acting antiviral.
