# Supplementary material for: Estimating the causal effect of treatment with direct-acting antivirals on kidney function among individuals with hepatitis C virus infection
Source: PLoS One. 2022 May 13;17(5):e0268478. doi: 10.1371/journal.pone.0268478 (PMC9106151; doi:10.1371/journal.pone.0268478)
Supplement: S5 Table — (DOCX) [file pone.0268478.s012.docx]

| **Characteristic** | **Received DAA within 3 months of baseline (N=854)** | **Did not receive DAA within 3 months of baseline (N=1321)** |
| --- | --- | --- |
| Age, years | 55 [46, 61] | 51 [36, 59] |
| Male | 557 (68%) | 880 (67%) |
| BMI, kg/m^2^ | 28 [25, 32] | 27 [24, 31] |
| Race/ethnicity  Black  Hispanic or Latino  White  Other^a^  Missing | 338 (40%)  131 (15%)  322 (38%)  23 (3%)  40 (4%) | 460 (35%)  224 (17%)  579 (44%)  26 (2%)  32 (2%) |
| Health insurance  Public  Private  Other^b^ | 529 (62%)  104 (12%)  221 (26%) | 942 (71%)  84 (6%)  295 (23%) |
| Level of education  Less than high school  HS degree or more  Missing | 323 (38%)  409 (48%)  122 (14%) | 524 (40%)  605 (46%)  192 (14%) |
| ALT, U/L | 55 [36, 95] | 53 [31, 92] |
| AST, U/L | 51 [35, 81] | 49 [32, 84] |
| Platelet count, k/uL | 202 [153, 249] | 208 [162, 259] |
| eGFR, ml/min/1.73m^2^ | 96 [82, 106] | 99 [84, 111] |
| Fibrosis Stage from Fibroscan  F0 – F2  F3 – F4  Missing | 363 (42%)  255 (30%)  236 (28%) | 311 (24%)  138 (10%)  872 (66%) |
| Fibrosis-4 Score Category  <1.45 (no significant fibrosis)  1.45-3.25 (non-cirrhotic fibrosis)  >3.25 (cirrhosis) | 336 (39%)  305 (36%)  213 (25%) | 588 (44%)  457 (35%)  276 (21%) |
| Diabetes | 168 (20%) | 216 (16%) |
| Hypertension | 520 (61%) | 735 (56%) |
| Diagnosis of Drug Use Disorder | 210 (25%) | 664 (50%) |
| Diagnosis of Alcohol Use Disorder | 52 (6%) | 168 (13%) |
| Mental Illness | 78 (9%) | 172 (13%) |
| HIV co-infection | 56 (7%) | 152 (12%) |

BMI, body mass index. ALT, alanine transaminase. AST, aspartate transaminase. Median [interquartile range] or frequency (percentage) presented.
^a^Other means American-Indian/Native American, Asian, Native Hawaiian/Pacific Islander, or other Pacific Islander.
^b^Other means such as charity or self-pay.
