# Supplementary material for: Estimating the causal effect of treatment with direct-acting antivirals on kidney function among individuals with hepatitis C virus infection
Source: PLoS One. 2022 May 13;17(5):e0268478. doi: 10.1371/journal.pone.0268478 (PMC9106151; doi:10.1371/journal.pone.0268478)
Supplement: S7 Table — (DOCX) [file pone.0268478.s014.docx]

| **Inclusion criteria** | **Hypertension** | **Intervention** | **Estimate^a^ (95% CI)** | **Estimated difference (95% CI)** |
| --- | --- | --- | --- | --- |
| Cohort 1^b^ | Yes (N=760) | No DAA initiation | 7% (4, 19) | 0 (Ref.) |
|  |  | DAA initiation | 6% (4, 16) | -1% (-6; 2) |
|  | No (N=682) | No DAA initiation | 5% (3, 9) | 0 (Ref.) |
|  |  | DAA initiation | 3% (2, 6) | -2% (-6; 1) |
| Cohort 2^c^ | Yes (N=495) | No DAA initiation | 5 (-1, 12) ml/min/1.73m^2^ | 0 (Ref.) |
|  |  | DAA initiation | -1 (-5, 2) ml/min/1.73m^2^ | -6 (-12, 0) ml/min/1.73m^2^ |
|  | No (N=238) | No DAA initiation | 9 (-15, 23) ml/min/1.73m^2^ | 0 (Ref.) |
|  |  | DAA initiation | 7 (-12, 16) ml/min/1.73m^2^ | -2 (-12, 9) ml/min/1.73m^2^ |

CI, confidence interval.
^a^For cohort 1, we estimate risk of Stage 3 CKD using the parametric g-formula. For cohort 2 we estimate mean change from baseline eGFR using the parametric g-formula.
^b^Normal kidney function defined as eGFR>90 ml/min/1.73m^2^.
^c^CKD Stage 2-4 defined as 90≥eGFR>15 ml/min/1.73m^2^.
